# Mucorales Species and Macrophages

**DOI:** 10.3390/jof6020094

**Published:** 2020-06-26

**Authors:** Francisco E. Nicolás, Laura Murcia, Eusebio Navarro, María Isabel Navarro-Mendoza, Carlos Pérez-Arques, Victoriano Garre

**Affiliations:** Departamento de Genética y Microbiología, Facultad de Biología, Universidad de Murcia, 30100 Murcia, Spain; fnicolas@um.es (F.E.N.); lauramur@um.es (L.M.); sebi@um.es (E.N.); mariaisabel.navarro3@um.es (M.I.N.-M.); carlos.perez6@um.es (C.P.-A.)

**Keywords:** phagosome maturation, RNAi, germination, apoptosis, melanin, iron, nutritional immunity

## Abstract

Mucormycosis is an emerging fungal infection caused by Mucorales with an unacceptable high mortality rate. Mucorales is a complex fungal group, including eleven different genera that can infect humans. This heterogeneity is associated with species-specific invasion pathways and responses to the host defense mechanisms. The host innate immune system plays a major role in preventing Mucorales growth and host invasion. In this system, macrophages are the main immune effector cells in controlling these fungi by rapid and efficient phagocytosis of the spores. However, Mucorales have evolved mechanisms to block phagosomal maturation and species-specific mechanisms to either survive as dormant spores inside the macrophage, as *Rhizopus* species, or geminate and escape, as *Mucor* species. Classical fungal models of mucormycosis, mostly *Rhizopus*, have made important contributions to elucidate key aspects of the interaction between Mucorales and macrophages, but they lack robust tools for genetic manipulation. The recent introduction of the genetically tractable *Mucor circinelloides* as a model of mucormycosis offers the possibility to analyze gene function. This has allowed the identification of regulatory pathways that control the fungal response to phagocytosis, including a non-canonical RNAi pathway (NCRIP) that regulates the expression of most genes regulated by phagocytosis.

## 1. Introduction.

Fungal spores are ubiquitous threats to human health that cause diverse pathologies ranging from mild and treatable allergic diseases to lethal infections. Fungi colonize both indoor and outdoor environments, and their spores pullulate at levels 1000-fold higher than other common airborne allergens [1], making the interaction between fungal pathogens and human hosts virtually inescapable. In this scenario, evolution has forced the development of molecular mechanisms to both attack fungal pathogens and defend the barrier surfaces in the hosts [2]. In these barrier surfaces (skin, pulmonary epithelium, and mucous membranes), the recruitment of the innate immune cells is highly effective to protect humans from thousands of fungal species.

The regular functioning of the innate immune system is sufficiently efficient to inactivate most of the fungal threats that are continuously encountering the barrier surfaces of the host. However, fungal infections represent a serious concern for immunocompromised individuals who must entirely rely on antifungal treatments. In this sense, this review focuses on the fungal order Mucorales, an ancient group of fungi that comply with both an opportunistic preference for immunocompromised hosts [3] and a natural antifungal resistant to most of the compounds regularly used for other fungal groups [4]. Once Mucorales overcome the innate immune system, they establish an infection known as mucormycosis, a highly lethal disease that presents an overall mortality rate of around 50% [5]. Mucorales present specific features such as coenocytic hyphae and a cell wall containing polysaccharide chitosan (an N-deacetylated version of chitin), whereas other distant groups of fungi like Ascomycetes and Basidiomycetes have septate hyphae with chitin as the main polysaccharide [6]. Nonetheless, there are no studies directly relating these specific features of Mucorales to their intrinsic antifungal resistance and high mortality rates. More recent studies have found new molecular particularities of Mucorales that could partially explain the antifungal resistance observed in these fungi [7]. This is the case of the ergosterol synthesis system, a frequent target for many of the current antifungal compounds. In this system, the lanosterol 14α-demethylase CYP51 F5 presents two amino acid substitutions conserved only in Mucorales, which could explain their intrinsic antifungal resistance to short-tailed azole compounds [7]. Another specific feature of Mucorales related to their antifungal resistance is a unique mechanism for the generation of epimutants [8,9]. These epimutants are strains temporarily adapted to the presence of different antifungal compounds, showing an acquired resistance that is lost once the compound disappears. The mechanism for this acquired resistance is based on the RNA interference (RNAi) mechanism of these fungi, which specifically destroys the mRNA of the gene that encodes the corresponding protein targeted by the antifungal compound [8,10,11].

The resistant of Mucorales to antifungals comes along with a reduced knowledge on the interaction of the host phagocytic cells with the spore and its implications in the pathophysiology of mucormycosis. This review will summarize current information related to macrophage–Mucorales interactions, describing and discussing new advances and future perspectives.

## 2. Mucoralean Species Involved in Mucormycosis

The order Mucorales is a group of ancient fungi classified in the subphylum Mucoromycotina. They are commonly known as “early-diverging fungi” because they are in a basal position with respect to Basidiomycota and Ascomycota in the fungal tree of life [12]. Last reports delimitate the order Mucorales to 55 genera and 260 species [13], including 25 species from 11 genera that cause infections in humans [14]. The most prevalent genus causing mucormycosis is *Rhizopus*, followed by the genera *Mucor*, *Lichtheimia*, and *Rhizomucor* [5,15,16]. Other less frequent pathogenic species can be found in the genera *Syncephalastrum*, *Cunninghamella*, *Apophysomyces*, and *Saksenaea* [17]. Traditionally, mucormycosis is defined generally as a fungal infection caused by any species of the order Mucorales, characterized by accelerated tissue invasion and necrosis followed by angioinvasion and dissemination. However, recent studies are unveiling particularities of this disease, depending on the fungal species that cause the infection. For instance, mucormycosis can manifest as a cutaneous, rinocerebral, or pulmonary infection, which recently has been related to the infecting species that is involved and the route of infection [15]. In this sense, Mucorales can be classified attending to the primary route of infection: either airborne or by contact. *Rhizopus* and *Rhizomucor* are primarily airborne, whereas *Mucor* and *Lichtheimia* infect the hosts mostly by contact [15]. This preference for the route of infection can be explained by the differences found in the sporangia of these genera [15]. Thus, *Rhizopus* and *Rhizomucor* present dry sporangia over long and thin sporangiophores fully adapted to release the spores in the air. On the contrary, *Mucor* and *Lichtheimia* generate wet sporangia that release the spores in small droplets when several sporangia stick together. Nonetheless, dried mass of spores can also be dispersed by air using the airborne route as a secondary way of infection [15]. These considerations might explain the prevalence of *Mucor* and *Lichtheimia* species in infected burn wounds and trauma injuries, usually caused by contact with soil or contaminated surfaces [15]. Another important feature of Mucorales directly related to their ability to produce mucormycosis is their differential thermotolerance. *Rhizopus* and *Lichtheimia* are thermotolerant and can grow at 37 °C [18,19], *Rhizomucor* species are thermophilic and can grow up to 55 °C [20], and *Mucor* species are usually mesophilic [21]. All the mucoralean strains causing mucormycosis in humans must be adapted to grow at 37 °C, which means that species isolated from patients that do not belong to the genera *Rhizopus* and *Lichtheimia* must present additional molecular adaptations to allow germination inside the phagocytic cells of the host. These molecular adaptations and the mechanisms to overcome the fungicidal attack of macrophages are widely uncharacterized in Mucorales, and their future unveiling will uncover the pathways that different mucoralean species chose to produce mucormycosis.

## 3. Host Defense against Mucorales

Mucormycosis is initiated by asexual resting spores that get in contact with skin or mucosa. Tissue invasion is largely prevented by physical barriers of the innate immune system, but a defective function of the immune cell effectors or damage of the skin or mucosa may result in mucormycosis. Lungs deserve special attention because they are the main entrance of the spores in the body and are equipped with specialized macrophages, alveolar macrophages, which are in the airway lumen in close contact with the respiratory epithelium [22]. These specialized macrophages and other macrophages play a pivotal role, with other innate immune cells having a minor role. Here, we outline the most recent signs of progress in the function of the different cells involved in the defense against Mucorales. The detailed function of these cells can be found in recent extensive reviews [23,24,25].

### 3.1. Epithelial Cells

Epithelial cells make up an external barrier of defense against any type of infection, including mucormycosis. Indeed, lesions in their integrity are considered as one of the risk factors of Mucorales infection [26]. Studies performed in *Rhizopus oryzae* revealed that the spore adhesion to the basement membrane proteins exposed after epithelial damage is the initial step to active spore germination and host infection [27]. Interaction between Mucorales spores and epithelial cells has been studied frequently in vitro using the alveolar epithelial cell line A-549 [28,29] and also in vivo [29]. Analyses of the host transcription response to Mucorales revealed the activation of several signaling pathways well known as part of the host response to fungal pathogens, including tumor necrosis factor, interleukin-1 (IL-1) alpha and beta, nuclear factor kappa B, mitogen-activated protein kinase, IL-22, and IL-17A [28,29]. In addition, an initial analysis suggested that the platelet-derived growth factor receptor B (PDGFRB) signaling is used by Mucorales as a mechanism to damage barrier host cells because its inhibition reduces epithelial damage [28]. Interestingly, a second transcriptomic analysis unveiled the activation of epidermal growth factor receptor (EGFR) signaling in alveolar epithelial cells, which is phosphorylated upon in vitro infection of these cells with *Rhizopus delemar* spores. It is postulated that EGFR signaling promotes the disease, as specific inhibition of this pathway with cetuximab or gefitinib reduces invasion and damage in the airway epithelial cells. Moreover, in vivo experiments showed that gefitinib treatment increases the survival of infected mice [29].

### 3.2. Phagocytes

Neutrophils and macrophages are essential components of an early immune response in fungal killing [30] promptly recruited in the point of infection in Mucorales infection [31,32]. Macrophages play a pivotal role in the defense against these fungi because their specific depletion in larval zebrafish and mouse models results in a higher susceptibility to infection [32,33]. Analysis of the kinetics of recruitment and degree of association with immune cells in immunocompetent mice infected intratracheally with *R. oryzae* revealed that, although a significant influx of neutrophils in the lungs, most of the spores were associated with alveolar and interstitial macrophages and dendritic cells. Confocal images in sorted cells showed that the spores were predominantly phagocytosed by alveolar macrophages followed by interstitial macrophages, highlighting the importance of alveolar macrophages in lung infection [32]. This study and others have shown that healthy alveolar macrophages fail to kill *Rhizopus* resting spores, although they can produce spore clearance of swollen and active spores [34,35]. Despite the inability to kill resting *Rhizopus* spores, alveolar macrophages from healthy mouse and rats block germination [36,37]. In rats, the inhibition of *Rhizopus* spore germination by alveolar macrophages is mediated by the generation of nitric oxide from oxidation of L-arginine, but the mechanism remains poorly understood [37]. In addition, phagocytosis in macrophage activates an iron restriction response as a part of the nutritional immunity [33]. Other Mucorales, exampled by *Mucor* species, probably also confront nutritional stress in the phagosome (see below), but they are able to overcome this defense mechanism and germinate inside the macrophage in vitro [38,39,40].

Neutrophils are important in host defense against Mucorales, considering that neutropenia is a risk factor for mucormycosis [41,42]. These cells may cause both spore and hyphae damage by releasing reactive oxygen metabolites, cationic peptides, and perforin. Neutrophil-spore interaction leads to the expression of toll-like receptors and the production of necrosis factor (TNF)-α, interferon (IFN)-γ, and IL-1b that facilitate the activation of other immune cells [43]. Besides the spore developmental stage or the hots immunocompetence, phagocytosis by neutrophils can be altered by other factors such as hyperglycemia, acidosis, and high iron levels that may be contributed to Mucorales infection [44,45].

In addition to the mechanisms described above, phagocytes can restrict the spores to the point of the infection by forming tight cell clusters around spores resembling early granulomas [33,46]. Spores are contained in these granulomas, where there is a lack of reactive oxygen burst, and failure to kill fungal spores as mucormycosis was reactivated by dexamethasone-induced immunosuppression [46].

### 3.3. Natural Killer Cells

Natural killer (NK) cells play a role in the immunosuppression of Mucorales infection by hyphae damage. The interaction between hyphae and NK cells induces their activation and perforin release [47]. This response is generalized among Mucorales species but only for the fungal hyphae and does not affect spores [48]. Nevertheless, studies in *R. oryzae* showed that hyphae have an immunosuppressive effect because hyphae can inhibit the NK cell secretion of immunomodulatory chemokines IFN-γ and RANTES (Regulated upon Activation, Normal T Cell Expressed and Presumably Secreted) protein [47,49].

### 3.4. Platelets

Recent studies reported that, beyond the homeostasis function, platelets have antimicrobial and antifungal properties. Platelets inhibit spore germination and hyphal grow in *Rhizopus, Rhizomucor, Mucor*, and *Absidia* species via granular secretion of proinflammatory and anti-inflammatory cytokines and chemokines. Besides, platelets bind to other cells to produce phagocytosis and dendritic cells and B and T lymphocyte activation [48,50,51].

### 3.5. Dendritic and Lymphocyte T Cells

The adaptative immune response during Mucorales infection has been poorly studied. Nevertheless, recent reports highlight a higher role of this specific immune system. Among components of the adaptative immune system, T cells have an essential role against mucormycosis. The presence of Mucorales hyphae induces CD4^+^ and CD8^+^ T cell production that release cytokines such as IL-4, IFN-γ, IL-10, and IL-17, and mediate hyphal damage [52]. Consequently, lymphopenia has been associated with increase mortality in pulmonary mucormycosis and hematological malignancies [53,54]. Moreover, dendritic cells can trigger T cell response playing as intermediate cells between the innate and adaptative responses [55]. The presence of *R. oryzae* hyphae actives IL-23 production by dendritic cells, inducing T helper 17 cells (Th-17). Th-17 cells secreted IL-17 promoting neutrophils response [56]. 

## 4. The Responses of Mucorales Spores to Phagocytosis

Host innate immune cells display many available arms to block the fungal invasion, but fungi have evolved strategies to counteract this attack, which is also true for Mucorales. Despite that macrophages and neutrophils are rapidly recruited to the infection point in mammals and zebrafish infection models [30,31,33], Mucorales spores are phagocytized predominantly by macrophages [32]. Therefore, numerous works have analyzed the responses of the engulfed spores to the phagocytosis, where different types of macrophages have been analyzed (Table 1), including alveolar macrophages [32], bone marrow-derived macrophages (BMDMs) [32], murine alveolar cell lines [34], and murine macrophage cell lines [57,58]. Many fungal pathogens, including Mucorales, have evolved mechanisms to avoid or survive the macrophage confrontation following different strategies (Table 1) [30]. A critical point in fungal invasion is the germination to produce hyphae that cannot be engulfed by the macrophages because of their size [30]. Therefore, macrophages rapidly engulf fungal spores once they get in contact with the host, but they are unable to kill Mucorales spores, resulting in two main species-specific outcomes. In some species, represented by *R. oryzae* and *R. delemar*, phagocytosis blocks germination of resting spores remaining viable inside alveolar macrophages in the lungs of immunocompetent mice at least for 10 days post-infection [32]. Mucorales spores inside macrophages have been observed also in human histopathology samples of surgical specimens from a patient with disseminated mucormycosis [32]. Persistence inside the macrophage may facilitate Mucorales spore dissemination, as has been suggested for *Cryptococcus neoformans* [59], and this could explain how spores of a *L. corymbifera* virulent strain are more readily phagocytosed than those of attenuated strains [34].

**Figure 1:**
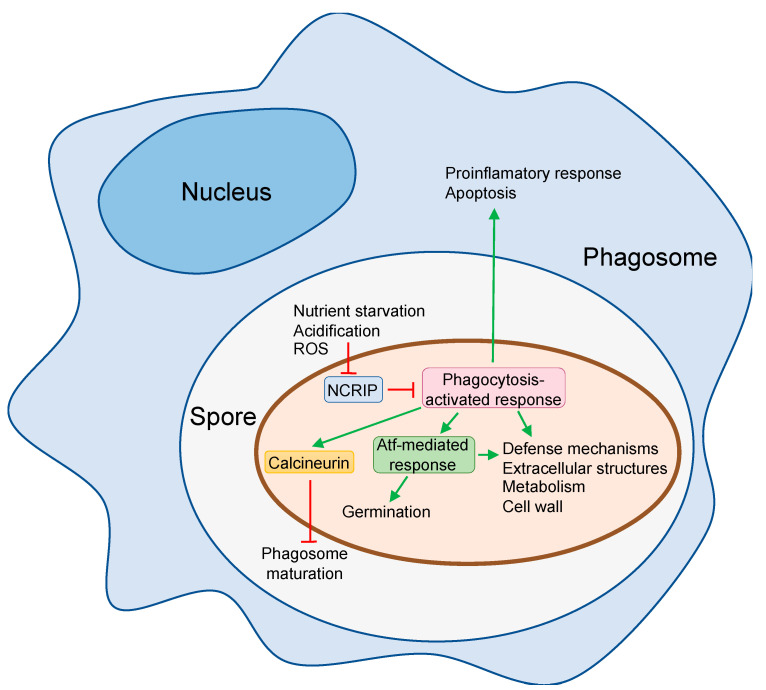
Mechanisms of *Mucor circinelloides* to escape from macrophage fungistatic activity. When *M. circinelloides* spores are phagocytized, a complex gene response is activated to survive macrophages attack that encompasses the differential expression of genes involved in defense mechanisms, extracellular structures, metabolism, and cell wall [57]. This response also up-regulates transcription factors *atf1* and *atf2* that regulate germination at low pH and other processes involved in the response to phagocytosis [57]. Calcineurin pathway is also activated and promotes phagosome maturation arrest [63]. The spore response to phagocytosis also comprises the activation of macrophage genes involved in apoptosis and proinflammatory response [57]. The NCRIP represses most of the genes of the phagocytosis-activated response in the absence of macrophages [58], suggesting that some of the signals present in the phagosome repress this RNAi-related pathway to allow the activation of the spore response to phagocytosis. Green arrow, activation; red T, repression.

Other Mucorales, typified by *Mucor* species, are also resistant to phagocytosis, but in this case, they can germinate inside the phagosome and kill macrophages in vitro and in vivo [31,38,39]. Infection of adult zebrafish with *M. circinelloides* spores induced cell death mediated by apoptosis in macrophages but not neutrophils [31]. Conversely, *R. oryzae* does not induce either apoptosis or necrosis of host cell during interaction in vitro with murine BMDMs [32]. However, it induces phagosome maturation arrest via targeting LC3-associated phagocytosis (LAP), which is a major antifungal pathway regulating early events in the biogenesis of *Aspergillus fumigatus* phagosome [61,62]. Phagosome maturation arrest is mediated by cell wall melanin [32]. Similarly, *Mucor* induces blockage of phagosome maturation in a murine macrophage cell line and BMDMs [39], but in this case, it depends on the calcineurin signal pathway (Figure 1) [63].

Despite the evident proof that Mucorales can escape from the macrophages attack, their mechanisms are poorly characterized. The study of the host-pathogen interaction at a transcriptomic level may elucidate the regulatory pathways involved during phagocytosis. A transcriptomic analysis of the interaction between *R. delemar* (strain 99-880) and BMDMs revealed that the majority of genes previously implicated in the iron acquisition were up-regulated during infection, including *fet3* and *ftr1* [32]. These genes code for a multicopper ferroxidase and a high-affinity iron permease and comprise the major iron assimilation pathway of *Rhizopus* and *M. circinelloides*, which has a pivotal pathogenetic role during in vivo infection [60,64]. Reduction of *ftr1* expression in *R. delemar* and deletion of *fet3* in *M. circinelloides* compromised their ability to acquire iron and reduced their virulence in diabetic ketoacidotic (DKA) and immunocompromised mice, respectively [60,64]. Importantly, immunization with anti-Ftr1 immune sera protected DKA mice from infection with *R. delemar* [60].

Other transcriptome studies have focused on the characterization of mechanisms used by Mucorales species that are able to germinate and grow inside the macrophages. These species should be able to mount an active defense to grow in a harsh and poor environment as the phagosome. The transcriptomic response to phagocytosis has been studied using virulent and attenuated strains of *M. circinelloides,* followed by the study of gene function by the generation of knockout mutants [57]. This analysis identified a large number of genes (>9% of total genes) differentially expressed in the two *M. circinelloides* pathotypes after 5 h of interaction with a murine macrophage cell line, revealing a general response showed by both strains and a virulence-specific response. The general response comprises an enrichment in genes involved in nutrient assimilation and metabolism, suggesting that *M. circinelloides* can shift toward alternative nutrient sources to allow germination inside the phagosome. The virulence-specific response highlighted an increase in biological processes involved in regulating cAMP-dependent intracellular signal transduction pathways and the response to oxidative stress that could explain the *M. circinelloides* ability to survive macrophage attack [57]. Functional characterization of *M. circinelloides* response to macrophages by deleting genes encoding transcription factors (*atf1*, *atf2*, and *gcn4*), extracellular proteins (*chi1* and *pps1*), and an aquaporin (*aqp1*) revealed that defects in most of the genes were associated with impaired germination inside the phagosome, which resulted in a decreased virulence in mice [57]. The two *atf1* and *atf2* genes code for two basic leucine zipper transcription factors similar to *Schizosaccharomyces pombe* Atf1, which is involved in oxidative stress [65]. The further transcriptomic analysis of the mutants in *atf1* and *atf2* suggested the existence of an Atf-mediated pathway that is involved not only in the response to oxidative stress but also in the macronutrient metabolism (Figure 1). In addition, this Atf-mediated pathway regulates germination at low pH because mutants in *atf1, atf2,* and in genes controlled by the pathway showed germination defects at acidic pH, suggesting that the pathway is activated by the low pH in the phagosome [57].

During mice infection, *M. circinelloides* undergoes a novel RNAi epimutation phenomenon that silences target genes, developing a transient an adaptive advantage to a hostile environment [66]. This novel epimutational mechanism of drug resistance interacts with a non-canonical RNAi pathway (NCRIP) [9]. The NCRIP regulates the mRNA levels of specific genes post-transcriptionally by degradation using a mechanism that includes RNA-dependent RNA polymerases (RdRPs) and a novel ribonuclease III-like named R3B2 [67]. This silencing pathway regulates multiple genes involved in fungal physiology that include resistance to oxidative stress, suggesting an active role in regulating virulent processes. The transcriptomic analysis of mutants lacking NCRIP activity during phagocytosis revealed an NCRIP-dependent virulent response [58]. Interestingly, the expression of *atf1, atf2,* and many of its secondary targets are controlled by the NCRIP, indicating that this novel pathway is involved in the response to environmental signals prompted by the phagosome, regulating downstream elements like the Atf-mediated response (Figure 1). Deeper analyses revealed that the NCRIP is constitutively active during vegetative growth, and the silencing mechanism control is released during phagocytosis. The interaction of the spores with macrophages is proposed to inhibit the NCRIP, resulting in the activation of the genetic program to overcome host defense mechanisms (Figure 1) [58]. The signals and the transduction pathways that regulate the NCRIP are unknown, but typical components of transduction signal pathways as heterotrimeric G proteins are required for full virulence in *M. circinelloides*. Genes *gpa11* and *gpa12* encoding α subunits of heterotrimeric G proteins show high transcript levels in resting spores [68] and are required to survive oxidative stress, phagocytosis and virulence in DKA mice [69]. Moreover, spores produced from cultures supplemented with native human blood serum show increased germination, resistance to oxidative stress, and survival to phagocytosis by macrophages, suggesting thermolabile components in the blood serum triggering a *M. circinelloides* response involved in macrophage interaction [70]. It is tempting to speculate the existence of a possible functional link connecting these independent observations in which the heterotrimeric G proteins would mediate the repression of the NCRIP in response to a blood serum component, but this hypothesis is still waiting to be explored.

## 5. Future Directions

Pharmacological control of mucormycosis faces the problem of the innate resistance of Mucorales to most of the available antifungal drugs. The improvement in the clinical treatments for other challenging diseases and the increasing aging population are raising the number of people susceptible to Mucorales infection. Therefore, it is imperative to develop new drugs to combat mucormycosis. The interaction between Mucorales and macrophages is a critical initial point of the invasion, and its characterization may provide targets for therapeutic treatments of the disease. Advances in recent years based on the application of “omics” technologies and the incorporation of models with robust molecular genetics have revealed some of the mechanisms used by these fungi to resist or overcome the macrophage attack. However, these advances represent only the tip of the iceberg, and most of the regulatory events that take place in the fungi during the phagocytosis are unknown. Future transcriptomic and proteomic analyses of the interaction between different relevant causing agents of mucormycosis are expected to reveal the signal transduction and regulatory pathways that control the general and species-specific mechanisms involved in the response of Mucorales spores to phagocytosis. The continuous use of genetically amenable models, such as *M. circinelloides*, and the adaptation of CRISPR/Cas technologies to most frequent and genetically untreatable pathogenic species producing mucormycosis are supposed to allow molecular dissection of Mucorales responses to phagocytosis in the near future, providing the long-awaited targets to create new antifungals.

## Figures and Tables

**Table 1:** Results of the interactions between macrophages and mucoralean species.

Host Cell	Mucoralean Species	Interactions	References
Alveolar macrophages and BMDMs	*R. oryzae* *R. delemar*	Macrophages fail to kill resting spores, but they inhibit spore germination via iron starvation. Spores block phagosome maturation via cell wall melanin.	[32,35,36,37]
Murine cell line and BMDMs	*M. circinelloides*	Macrophages fail to kill resting spore and do not inhibit spore germination. Spores block phagosome maturation via calcineurin signal pathway. Spores kill macrophages.	[38,39,40,60]
Murine alveolar cell line	*L. corymbifera*	A virulent strain shows increased phagocytosis	[34]
Zebrafish macrophages	*M. circinelloides*	Formation of early granulomas in vivo. Induction of macrophages apoptosis in vivo.	[31,33,46]
